# Systematic Hospital-Based Travel Screening to Assess Exposure to Zika Virus^1^

**DOI:** 10.3201/eid2602.190292

**Published:** 2020-02

**Authors:** Aftab Iqbal, Robert Colgrove, Vito Iacoviello, Barbra M. Blair, Lin H. Chen

**Affiliations:** Providence Community Health Centers, Providence, Rhode Island, USA (A. Iqbal);; Mount Auburn Hospital, Cambridge, Massachusetts, USA (A. Iqbal, R. Colgrove, V. Iacoviello, L.H. Chen);; Beth Israel Deaconess Medical Center, Boston, Massachusetts, USA (B.M. Blair);; Harvard Medical School, Boston (R. Colgrove, V. Iacoviello, B.M. Blair, L.H. Chen)

**Keywords:** Zika, screening, travel health, at-risk population, pregnancy, reproductive age, surveillance, vector-borne infections, mosquitoborne diseases, viruses, Zika virus, ZIKV, United States, *Suggested citation for this article:* Iqbal A, Colgrove R, Iacoviello V, Blair BM, Chen LH. Systematic hospital-based travel screening to assess exposure to Zika virus. Emerg Infect Dis. 2020 Feb [*date cited*]. https://doi.org/10.3201/eid2602.190292

## Abstract

We queried hospital patients about international travel in the previous 30 days to assess potential importation of emerging infections. We used 12 months of deidentified data to analyze patient demographics, travel destinations, and diagnoses for exposure to Zika virus. Our approach could be used to analyze potential infectious disease exposures.

Incidence of Zika virus (ZIKV) infections rose rapidly in early 2015, and local transmission was confirmed in 84 countries and territories by March 2017 (*1*). Although ZIKV typically causes mild symptoms (*2*,*3*), in utero infection can cause congenital Zika syndrome (*4*,*5*). The threat of in utero infection, along with sexual transmission (*6*,*7*), led to advisories for women who were pregnant, or might become pregnant, and their partners to avoid travel to countries or areas with ZIKV transmission (*7**–**10*).

After implementing reactive screening during several global infectious disease outbreaks, including the 2014 Ebola outbreak, Mount Auburn Hospital (Cambridge, Massachusetts, USA) incorporated a standardized screening question regarding international travel into all hospital visits beginning in September 2015. To detect potential travel-associated exposures, patients were asked, “Have you traveled outside of the U.S. within the past 30 days?” Each quarter during November 1, 2015–October 31, 2016, we aggregated deidentified patient data to estimate the proportion of patients with potential ZIKV exposure and the possibility for congenital Zika syndrome.

## The Study

During November 1–December 31, 2016, we retrospectively analyzed deidentified patient demographic, travel destination, and medical services data from the hospital database. We analyzed records from patients admitted as inpatients, and those seen in the emergency department/walk-in center (ED/WIC) and by other services during November 1, 2015–October 31, 2016. We included data from patients who responded “yes” to the travel screening question and provided a travel history and for whom diagnostic data were available. We categorized destination countries according to the World Health Organization 2016 classification for ZIKV transmission (*11*): category 1, countries that reported outbreaks from 2015 onward; category 2, countries with possible endemic transmission or evidence of local mosquitoborne ZIKV infections in 2016; and category 3, countries with evidence of local mosquitoborne ZIKV infections during or before 2015, but without documentation of cases in 2016, or designated as outbreak terminated. We defined reproductive age as 15–49 years of age for female patients and ≥15 years of age for male patients (*12*). We extracted records with International Classification of Diseases, 10th Revision, codes applicable to pregnancy, including Z33.1, Z34.91, Z34.92, Z34.93, and Z34.90, and diagnosis descriptions that met the Zika disease case definition (*3*), which includes fever, rash, arthralgia, conjunctivitis, complication of pregnancy, or Guillain-Barré syndrome. We performed analyses by using IBM SPSS Statistics 17.0 (IBM, https://www.ibm.com). The Mount Auburn Hospital Institutional Review Board determined the activity to be exempt from review and approval.

We identified 5,642 patients who reported travel <30 days before their hospital visit. Of 5,004 patients who had complete demographic, destination, and diagnostic data, 3,109 (62.1%) were female and 1,895 (37.9%) male; patients were 10 months–94 years of age. A total of 959 (19%) were evaluated in the ED/WIC, and 161 (3.2%) with recent travel were hospitalized. The most frequently visited destinations were Canada, the United Kingdom, and Mexico (Table 1).

**Table 1:** Countries visited most frequently in the previous 30 days by 5,004 travelers and Zika-affected countries visited by 1,570 travelers, Cambridge, Massachusetts, USA

Rank	All destinations visited			Destinations with ZIKV transmission*	
	Destination	No. (%) travelers		Destination	No. (%) travelers
1	Canada	778 (15.5)		Mexico	318 (20.2 )
2	United Kingdom	363 (7.2)		Dominican Republic	119 (7.5 )
3	Mexico	318 (6.4)		Aruba	102 (6.4 )
4	France	315 (6.3)		Brazil	85 (5.4 )
5	Italy	265 (5.3 )		Costa Rica	84 (5.3 )
6	Germany	227 (4.5 )		Puerto Rico	83 (5.2 )
7	China	200 (4.0 )		Bahamas	81 (5.1 )
8	Ireland	158 (3.2 )		Virgin Islands	57 (3.6 )
9	India	155 (3.1 )		Colombia	48 (3.1 )
10	Spain	150 (3.0 )		Cuba	45 (2.8 )
*According to the World Health Organization (*11*). ZIKV, Zika virus.					

Among female patients, 1,579 (50.8%) were of reproductive age and 176 (5.7%) were pregnant. Among male patients, 1,850 (97.6%) were of reproductive age. Overall, 475 (30%) female and 514 (28%) male patients of reproductive age traveled to countries with ZIKV transmission. Of 176 pregnant women, 38 (22%) had traveled to countries with ZIKV transmission within the previous 30 days; 21 were in the first trimester (Figure).

**Figure:**
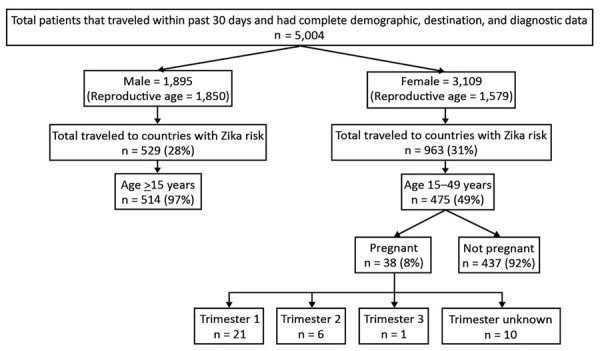
Flowchart of possible Zika virus exposure based on travel destination, sex, and pregnancy status in patients responding to a question on international travel <30 days before seeking care at a hospital, Cambridge, Massachusetts, USA.

When analyzed for destinations in WHO categories 1–3 (*11*), 1,492 travelers, 963 female and 529 male, visited 1,570 destinations with ZIKV transmission. Mexico, the Dominican Republic, Aruba, Brazil, and Costa Rica (Table 1) were the most frequently visited destinations, and the Caribbean was the most frequently visited region (n = 648).

Among patients who traveled to countries with ZIKV transmission whose admitting diagnosis or description was available, 42 listed symptoms compatible with ZIKV infection (Table 2). We did not identify Guillain-Barré syndrome or any complications of pregnancy among the 42 patients, but 2 had laboratory-confirmed ZIKV infection.

**Table 2:** Results of search for symptoms compatible with Zika virus clinical criteria in a study of travel screening for Zika virus infection, Cambridge, Massachusetts, USA*

Patient age, y/sex	Date of hospital visit	Destinations visited	Diagnosis and symptoms at admission
40/F	2015 Nov	Malaysia	Rash and other nonspecific skin eruption
49/F	2015 Nov	Thailand	Pruritus, unspecified; rash and other nonspecific skin eruption
59/F	2015 Nov	Malaysia, Indonesia	Fever, unspecified
15/F	2015 Nov	Costa Rica	Fever, unspecified; rash and other nonspecific skin eruption; myalgia
29/M	2015 Nov	Mexico	Unidentified influenza virus with other respiratory manifestations; fever, unspecified
5/M	2015 Dec	Brazil	Viral infection, unspecified; rash and other nonspecific skin eruption; fever, unspecified
48/M	2015 Dec	Mexico	Acute pharyngitis, unspecified; rash and other nonspecific skin eruption
40/M	2015 Dec	Mexico	Acute pharyngitis, unspecified; fever, unspecified
28/F	2016 Jan	Indonesia	Fever, unspecified; acute pharyngitis, unspecified; cough; myalgia
34/F	2016 Jan	The Bahamas	Viral infection, unspecified; fever, unspecified
61/F	2016 Jan	Mexico	Rash and other nonspecific skin eruption
39/F	2016 Jan	Argentina	Rash and other nonspecific skin eruption
1/M	2016 Jan	Mexico	Rash and other nonspecific skin eruption; fever, unspecified
33/M	2016 Jan	Colombia	Rash and other nonspecific skin eruption
55/M	2016 Jan	Costa Rica	Fever, unspecified; rash and other nonspecific skin eruption
58/F	2016 Feb	Mexico	Acute pharyngitis, unspecified; fever, unspecified; myalgia
UNK/M	2016 Feb	Brazil	Rash, cough, fever, sneezing; viral infection, unspecified
31/M	2016 Feb	Brazil	Viral infection, unspecified; rash and other nonspecific skin eruption
31/M	2016 Feb	Brazil	Posttravel follow-up; requested screening for other viral diseases; rash and other nonspecific skin eruption
28/F	2016 Mar	Costa Rica	Influenza-like symptoms; viral infection, unspecified
28/F	2016 Mar	Mexico	Rash and other nonspecific skin eruption
46/F	2016 Mar	Jamaica, Mexico	Influenza-like symptoms; cough; unidentified influenza virus with unspecified type of pneumonia
21/M	2016 Mar	Mexico	Influenza-like symptoms; viral infection, unspecified
32/M	2016 Mar	The Bahamas	Headache, back pain, vomiting; fever, unspecified; nausea with vomiting, unspecified
30/F	2016 Apr	Brazil	Fever and aches; viral infection, unspecified
69/F	2016 Apr	Haiti	Fever, unspecified
26/M	2016 Apr	Dominican Republic	Headache with rash; viral infection, unspecified
26/F	2016 May	Thailand	Fever, unspecified
39/F	2016 Jun	Mexico	Rash and other nonspecific skin eruption; nonvenomous insect bites on left and right upper arm
25/M	2016 Jun	Puerto Rico	Rash and other nonspecific skin eruption; other fatigue
44/F	2016 Jul	Dominican Republic	Rash and other nonspecific skin eruption
29/M	2016 Jul	Dominican Republic	Influenza-like symptoms Viral infection, unspecified
15/M	2016 Jul	Trinidad and Tobago	Rash and other nonspecific skin eruption Fever, unspecified; acute pharyngitis, unspecified
44/F	2016 Aug	Nicaragua	Rash and other nonspecific skin eruption
51/F	2016 Aug	Dominican Republic	Cough; fever, unspecified
51/M	2016 Aug	Malaysia, Turkey	Cough; fever, unspecified
36/M	2016 Aug	Brazil	Posttravel screening; fever, unspecified; unspecified conjunctivitis
28/F	2016 Sep	Chile	Influenza-like symptoms
34/F	2016 Sep	Indonesia	Acute pharyngitis, unspecified; fever, unspecified
28/F	2016 Sep	Virgin Islands	Rash; unspecified viral infection
39/M	2016 Sep	Dominican Republic	Rash and other nonspecific skin eruption
30/M	2016 Sep	Colombia	Possible Zika virus; patient was ill after travel
*Symptoms include rash, fever, conjunctivitis, arthralgia or myalgia, complications in pregnancy, or Guillain-Barré syndrome. Patients were asked, “Have you traveled outside the U.S. in the past 30 days?” UNK, unknown.			

Our results approximate the Global Travel Epidemiology Network analysis of pretravel consultations (*13*), in which 28% of 22,736 travelers planned trips to ZIKV-affected countries and >75% were of reproductive age. Another study retrospectively reviewed 46 patients for possible ZIKV infection and found 17% had laboratory evidence of infection (*14*). Applying this seropositivity rate to our study, if testing been done, 7 patients with symptoms compatible with ZIKV clinical criteria might have had laboratory-confirmed ZIKV infection. 

We found that a standardized question to screen for international travel provided a description of travel patterns for this patient community. Data from the 12-month period coincided with the rapid spread of ZIKV and revealed the sizable portion of patients who might have been exposed to ZIKV during travel. Population-based analysis of travel-related ZIKV exposure could provide estimates of at-risk populations and diagnostic testing needs, especially for pregnant women. The application is especially promising with newer electronic health record systems. However, limited testing capability might have underestimated the actual number of travel-associated cases (*15*), even when clinicians suspected ZIKV. 

Our study had some limitations. Because we only reviewed the population at 1 hospital during a single 12-month period, our results might not be generalizable to the US population. Our analysis relied on recent travel to countries and territories that reported ZIKV transmission, but some travelers might have visited risk-free settings, such as locations at higher altitudes, resulting in overestimation of the number of possible infections. Infectious ZIKV has been detected in semen mainly <30 days after fever onset, but its presence in semen has been documented longer (*10*,*15*); therefore, the potential number of ZIKV infections might exceed our estimate because sexual partners could become infected. Also, we did not have information on whether the patients of reproductive age were sexually active, fertile, had pregnant partners, or were planning conception. We might have missed cases for the following reasons: we relied on diagnoses and diagnostic descriptions, but omission of symptoms in these fields might not represent truly absent symptoms; some infected persons might have been unaware of ZIKV and might not have sought medical evaluation; the incubation period of sexually transmitted ZIKV might be >30 days and patients might have become ill after being seen; we did not collect or record ZIKV infections identified after the study period; only patients strongly suspected of ZIKV were tested due to limited laboratory capacity; and the travel screening question would not have identified sexually transmitted ZIKV infection in a patient who had not traveled internationally.

## Conclusions

We used a systematic travel screening question to analyze potential exposure to ZIKV in a hospital population. Because up to 80% of ZIKV infections are asymptomatic (*2*), we used travel to Zika-affected countries as a proxy for potential ZIKV exposure. In patients with international travel <30 days before seeking treatment, 31.4% visited countries with ZIKV transmission. Half of the female patients and most male patients were of reproductive age. In this population, 30% of female patients who were of reproductive age or pregnant reported travel with potential exposure to ZIKV; male patients similarly were affected. Despite severe restrictions on testing for ZIKV infection at the time of the study, our analysis demonstrated the ability to identify patients with clinical findings that fit the ZIKV case definition even if they were not tested. We also identified a large proportion of patients who should have received Zika pretravel counseling.

Analysis of the hospitalwide data for recent travel history provided a tool to assess the proportion of the population that might have been exposed to ZIKV. These data could inform population-based ZIKV vaccination needs in the future. In addition, systematic travel screening also could be applied to other imported emerging infections in the future.
